# Integrated decoys and effector traps: how to catch a plant pathogen

**DOI:** 10.1186/s12915-016-0235-8

**Published:** 2016-02-19

**Authors:** Jeffrey G. Ellis

**Affiliations:** CSIRO Agriculture, GPO Box 1600, Canberra, 2601 Australia

## Abstract

Plant immune receptors involved in disease resistance and crop protection are related to the animal Nod-like receptor (NLR) class, and recognise the virulence effectors of plant pathogens, whereby they arm the plant’s defensive response. Although plant NLRs mainly contain three protein domains, about 10 % of these receptors identified by extensive cross-plant species data base searches have now been shown to include novel and highly variable integrated domains, some of which have been shown to detect pathogen effectors by direct interaction. Sarris et al. have identified a large number of integrated domains that can be used to detect effector targets in host plant proteomes and identify unknown pathogen effectors.

Please see related Research article: Comparative analysis of plant immune receptor architectures uncovers host proteins likely targeted by pathogens, http://dx.doi.org/10.1186/s12915-016-0228-7

Since the time of writing, a closely related paper has been released: Kroj T, Chanclud E, Michel-Romiti C, Grand X, Morel J-B. Integration of decoy domains derived from protein targets of pathogen effectors into plant immune receptors is widespread. New Phytol. 2016 (ahead of print)

## A commonality between plant and animal innate immunity

Animal and plant innate immune systems share related cytosolic receptor proteins, the Nod-like receptors (NLRs), for detection of pathogens. It is often overlooked that plant NLRs are as important to human health as the animal NLRs by virtue of the link between health and good nutrition. Plant NLRs, by controlling plant diseases and the serious constraints these apply to food production, are in fact anti-starvation regulators of the human population. Consequently, NLR function is an intensely studied area in plant biology.

## Detecting effectors and the effects of effectors

NLRs in animals detect pathogen-associated molecular patterns (PAMPs) whereas in plants each, generally polymorphic, NLR recognises limited numbers of a large and highly diverse set of pathogen effector molecules that operate to obstruct the host plant’s immune response triggered by PAMPs via a non-NLR pathway (PAMP triggered immunity). Initially three domains were identified as the pillars of plant NLR protein architecture, an N-terminal signalling domain of one of two types, a central nucleotide binding “molecular switch domain” and a C-terminal leucine-rich repeat domain of ill-defined function that, in some NLRs, imparts specificity to the NLR–effector interaction. Additional “non-canonical” domains have also been identified in a limited number of functional plant NLRs [1, 2] and their crucial roles in effector detection have just been elucidated through experimentation [2, 3]. Critically, other plant proteins not carrying NLR motifs have domains that are related in sequence [2, 3]. So far a few of these non-canonical “integrated domains” (IDs) have been shown to act in the detection of pathogen effectors through effector–ID interaction and, in one case, ID modification by effector enzymatic function (see [3]). Other functions for IDs may emerge. Plant receptors carrying IDs are referred to by Saris et al. [4] as “NLR-IDs”, reflecting the fact that, in many cases, the functions of IDs are not yet elucidated. However, for those cases where the ID is related to known or presumed effector target sites in host proteins (virulence targets), a highly evocative metaphor has been coined [2, 3] referring to the non-canonical NLR domains as “integrated decoys”. Just recently one of these effector–integrated decoy interactions has been defined at the structural level after crystallisation of the interacting domains [5].

## A remarkable diversity of non-canonical integrated sequences in NLRs

Elucidation of the biology of four NLR-IDs has been one of the most exciting and novel recent advances in plant pathology. Consequently, researchers have begun to seek out and describe the diversity of the non-canonical domains of plant NLRs. The first report by Cesari et al. (see [2]) used bioinformatic methods to identify a large number of integrated protein domains in annotated plant NLRs. Now Sarris et al. [4] report the results of a huge and extensive search which has led to the identification of 14,363 NLRs in 40 plant genomes, of which 720 were found to carry 265 distinct integrated Pfam classes. At least 61 of these domains were present in NLRs from more than one plant family, indicating a recurrent theme among integrations. Further, Saris et al. reported that, on average, 10 % of plant NLRs contain IDs. These statistics make quite an impressive set of numbers. The majority of IDs occur as N- or C-terminal fusions with NLRs and the diversity includes integrations of two different domains in the same protein, sometimes at both ends. In a minority, including the rice NLR Pik-1, integration has occurred between the N-terminal signalling domain and the central nucleotide binding domain of the NLR (see [2]).

## What are the ID sequences and where do they come from?

A feature of IDs that is not obvious from the initial publications is the proportion of overlap between IDs and their closest conspecific non-NLR homolog, i.e., what proportion of the presumed ID donor protein is integrated? Also, what is the level of amino acid sequence identity between ID and closest non-NLR homolog? In brief, BlastP searches indicate matches below 70 % amino acid identity and alignments of 25–60 % to the ‘full length’ closest conspecific protein using RRS1 and RGA5 IDs in searches.

The underlying proposition of the integrated decoy hypothesis, and I am adding some of my own embellishments here, is that, through evolution, integration of a domain from an effector target or non-integrated effector decoy (*sensu* Kamoun and van der Hoorn [6]) has occurred in some NLRs and has been selected as an effective detector of pathogen infection. How this ‘improves’ the effectiveness of NLRs and non-integrated effector targets is open to conjecture. The rather low level of relationship in terms of relative size and amino acid sequence between IDs and related conspecific proteins has several implications. First, only the domain of the host target directly involved in effector interaction (and not function of the domain donor protein) is commonly integrated. Second, the lack of exact sequence match may mean that the original target is no longer present in the host genome or, more likely, in the absence of sequence constraint for function apart from effector binding or effector modification, the ID may have evolved to maximise interaction strength with the corresponding effector and, in addition, the ability to interact with several different effectors that share a single or even closely related family of effector targets in the host. This is certainly the case with RGA5 and Pik-1 in rice where the two diverged ID sequences interact with three amino acid sequence-unrelated effectors from a single pathogen species (see [2]). Interestingly these three effectors share very similar protein structure [7]. The WRKY domain in RRS1 also interacts with two sequence-unrelated effectors from two bacterial species, although the structures of both proteins have not been determined yet [3]. Significantly, the directly interacting IDs in Pik-1 and Avr-Pik are represented by alleles in host and pathogen undergoing an ‘arms race’ [2].

One further interesting point among NLR-IDs with known resistance function is provided by the rice Pi5-2 protein, aka Pii, which carries a C-terminal ID related to “AvrRpt2 cleavage site” related domain (see [2]), also found in several other monocot and dicot NLR-IDs [5]. The corresponding effector is Avr-Pii [2], whose host target, necessary for Pi5/Pii-AvrPii recognition, is a non-ID Exo70 protein [8]. Interestingly, Exo70 occurs as an ID in several grass species but not rice [2, 4].

## Contributions of “big biology”

The scale and dimension of bioinformatic discoveries challenge the experimental elucidation of the biological role of such a multitude. However, comparison of IDs detected bioinformatically by Sarris et al. [4] with a set of predicted (and some confirmed) *Arabidopsis* effector targets detected by a totally independent approach showed a 20 % overlap, consistent with the IDs representing effector targets. The *Arabidopsis* presumptive effector target panoply was detected by massive yeast two-hybrid screens for interaction between 8000 host proteins and a large number of known and predicted pathogen effectors from bacteria, oomycetes and fungi [9, 10]. A similar “big biology” approach of massively screening for interaction between all identified *Arabidopsis* NLRs, especially those carrying IDs, and the same set of pathogen (putative) effectors used above would provide an intriguing exercise in biological “cross verification” if the two approaches again lead to substantial overlap in the identified host proteins.

## Well-matched couples

Many known plant NLRs function singly to detect and respond to pathogens and so far these are all NLRs and not NLR-IDs. In fact, a critical aspect of well-studied NLR-IDs is that so far they all function as interacting protein pairs with a corresponding NLR partner (without an integrated domain) [1–3]. One hypothesis is that the integration of non-canonical sequences is incompatible with important NLR functions and the second NLR member of the pair is required to complement the compromised functions of the NLR-ID. In those pairs studied, it has indeed been observed that one of the pair acts as the pathogen sensor and the second as the trigger of defence activation. An intriguing question is whether this will be a universal feature of all the new NLR-ID proteins [4]?

## Synthetic disease resistance for crop protection?

**Fig. 1:**
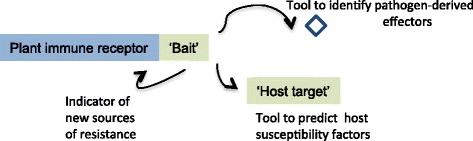
A diagram from Figure 6 of Sarris et al. [4] indicating what can be learned from “integrated domains” The intriguing question is whether “host targets” that have been (or will be) identified and not present in naturally occurring NLR-IDs could be integrated *in vitro* to make novel synthetic NLR-ID proteins for disease control in crops?

In their final summation (Fig. 1), Sarris et al. [4] “hypothesize that NLR-IDs provide clues to the host proteins targeted by pathogens” and this is indeed an exciting prospect in the field. The second point they hypothesise is that “this information can be deployed to discover new sources of disease resistance.” Cesari et al. (see [2]) and Maqbool et al. [5], respectively, have provided forthright insights into how this could be achieved by “R (NLR) protein engineering allowing effector targets to be fused to a receptor NLR….to create an effector trap” and determining whether “transferring unconventional integrated domains to different positions within and between NLRs will determine the importance of domain location, and whether these positions can accommodate novel integrated domains with the potential to deliver new-to-nature resistance capabilities.” Archimedes is claimed to have said “give me a lever long enough and a fulcrum on which to place it, and I shall move the world.” Perhaps with respect to NLR-IDs he would have said “give me a bag full of effector binding domains and a functional place to stick them in NLRs and I shall make a heap of synthetic plant disease resistance genes.” I am impatient for the first results.
